# Effects of thermal treatments on characteristics and morphological variations in the deposits of urea-SCR systems

**DOI:** 10.1007/s11356-021-14057-4

**Published:** 2021-06-01

**Authors:** Sadashiva Prabhu S, Kapilan Natesan, Nagaraj Shivappa Nayak

**Affiliations:** 1 Department of Mechanical and Manufacturing Engineering, Manipal Institute of Technology, Manipal Academy of Higher Education, Manipal, Karnataka 576104 India; 2 Department of Mechanical Engineering, Nitte Meenakshi Institute of Technology, P.B.No.6429, Yelahanka, Bangalore, Karnataka 560064 India; 3 KBAT Technologies Private Limited, Pune, Maharashtra 411033 India

**Keywords:** NO_x_, Selective catalytic reduction, Urea, Deposits, Thermal treatment, Morphology

## Abstract

Selective catalytic reduction (SCR) systems are employed by automobile manufacturers for the abatement of environmental pollutants like oxides of nitrogen (NO_x_) emitted from exhaust gases of diesel engines. In SCR, the urea-water solution (UWS) is injected to exhaust gases in the form of a spray to generate the reducing agent NH_3_. Deposit formation at lower temperatures is a major concern with this technology. The deposits not only create backpressure but also leak NH_3_ to the environment as they deplete. It is very important to know the depletion characteristics of deposits formed at lower temperatures in order to assess the NH_3_ leakage to the environment when the engine exhaust gases attain higher temperatures. In the present work, deposits formed at a low-temperature range of 150–200°C for continuous run along with UWS injection were investigated. Additionally, they were aged at 300°C in the absence of UWS to check the variation in characteristics with the rise of temperature. By gravimetric analysis, it is inferred that the deposits formed at higher pre-age temperatures are less prone to depletion as the temperature increases. The elemental analysis using energy-dispersive X-ray spectroscopy (EDX) indicates slight variation in carbon, nitrogen and oxygen compositions for all the pre-age conditions. As an extended study, the byproducts at pre-age and post-age conditions were investigated through X-ray diffraction (XRD). The compounds like cyanuric acid (CYA) and biuret were not observed when pre-age samples were aged at 300°C. Instead, the compounds like ammelide, ammeline, triuret and melamine were observed. Scanning electron microscope (SEM) study revealed morphological changes in both pre-age and post-age samples. Further, the crystallinity variations were also observed for the changes in the heating cycles during deposit formation. The gravimetric analysis of deposits in pre-age and post-age conditions helps in predicting the amount of deposits for transient load cycles.

**Supplementary Information:**

The online version contains supplementary material available at 10.1007/s11356-021-14057-4.

## Introduction

Diesel engines of heavy-duty automobiles contribute excess oxides of nitrogen (NO_x_) and their related health problems in almost all regions (Anenberg et al. 2017; Stohl et al. 2015). The mitigation of environmental pollutants like NO_x_ from the diesel engine exhaust is a secondary NO_x_ removal technique by aftertreatment (Baleta et al. 2016). Selective catalytic reduction (SCR) is the latest technology adopted by many automobile manufacturers to mitigate NO_x_, wherein ammonia (NH_3_) is used as a reducing agent (Baleta et al. 2015; Koebel and Strutz 2003). The reduction of NO_x_ takes place over the catalyst through various favorable reactions (Radojevic 1998; Fang and DaCosta 2003). In SCR, injected urea-water solution (UWS) evaporates and transforms into solid urea and water vapor. Solid urea undergoes thermolysis and generates NH_3_. But, at the low temperature of the exhaust gas, urea transforms into solid deposits. As urea deposits deplete, there will be changes in the stoichiometric NH_3_/NO_x_ ratio, which leads to NH_3_ slip to the environment when exhaust gas temperature increases. Some qualitative studies on deposits, and the precursors of deposits like wall film and entrainment from wall film are found in the literature (Varna et al. 2015; Shahariar and Lim 2018). The studies on various deposit byproducts, and factors affecting the deposit formation and its depletion are discussed in the following sections.

## Studies on urea deposit formation

### Urea deposit formation/depletion characteristics

Deposit formation is a time-dependent phenomenon in actual SCR systems. But, in the laboratory, accelerated tests are to be done to understand its behavior. Xu et al. (2007) generated deposits by dripping the catalyst with UWS and held at lower exhaust temperatures (<300°C). They also revealed that at temperatures below 150°C, most of the deposits are urea based which deplete completely below 250°C during aging. At about 300°C, the deposits contain cyanuric acid (CYA). With further increase in aging temperature, it was found that the majority of the deposits consisting of urea and CYA were vaporized above 350°C, and the decomposition depends mainly on the surface area of the catalyst (Xu et al. 2007).

Bai et al. (2014) revealed that urea and pyrolithic acids are the major components of the deposits by their thermogravimetric analysis (TGA) and Fourier transform infrared (FTIR) spectroscopy studies, and heating up to 500°C would reduce the quantity of deposits. The integrated injector mountings can prevent deposit formation effectively. Additionally, a rise in temperature and the correction in UWS dosage can reduce the urea deposits (Bai et al. 2014).

TGA studies also reveal that urea decomposition follows three stages (Zhang et al. 2017). In the first stage, i.e., T<130°C (below the melting temperature of urea), deposits are mainly of urea. For the second stage, i.e., between temperatures 130 and 190°C, a combination of urea, biuret and CYA was noticed. At the third stage, for temperatures above 190°C, CYA formed out of biuret, and above 200°C, the fast chemical transformation occurs through all the earlier processes. With a raised temperature of 250°C, ammelide formation was substantial, which is more stable than CYA (Zhang et al. 2017).

Another study on TGA of urea followed three steps. The first step corresponds to a 69% reduction in weight at temperature range 135–220°C with urea transforming to biuret. In the second stage (224–257°C), 13.3% reduction in weight was accounted for the formation of CYA and turning to NH_3_ and isocyanic acid (HNCO). In addition, ammelide was also considered to be existing in the second stage. In the third stage (307–372°C), 17.5% weight reduction was identified for the decomposition of CYA (Stradella and Argentero 1993).

A four-stage decomposition with weight losses of 66%, 13%, 18%, and 3% was recorded by the thermogravimetry/differential thermal analysis (TG/DTA) studies reveal that the heating rate is a significant factor for the decomposition of urea. After the first stage of decomposition, the polymerization and decomposition of remnant urea will be more predominant (Chen and Isa 1998).

The studies of Schaber et al. (2004) showed that the decomposition characteristics follow four stages. In the first stage up to 190°C, the urea decomposition continues, and Biuret reaches the maximum limit. Other products like CYA and ammelide form with kinetically slow reaction rates. In the second stage, i.e., 190–250°C, the decomposition of urea progresses, and biuret starts to decompose. This results in the increase of the production rate of CYA and ammelide and the appearance of ammeline and melamine in lesser percentages. In the third stage, i.e., 250–360°C, the remaining mass decomposes by sublimation. The fourth stage continues for final decomposition and elimination of remaining deposits (Schaber et al. 2004). By a sequence of acid and base hydrolyses, it was revealed that deposits at temperatures above 265°C contained a computable amount of oligomers and polymers (Eakle et al. 2016). The above studies reveal that UWS injection for exhaust gases between 130 and 300°C requires an additional effort to achieve the desired conversion efficiency.

### Factors affecting deposit formation during transient running condition

In the actual engine running conditions, the variables being transient, the overall effect may be different. The transient operating parameters like mass flow rate of exhaust gas, NO_x_ content, temperature of exhaust gas, ambient temperature, UWS spray properties, and UWS dosage have their effect on deposit formation. Further, the design parameters such as the position of urea injector, mounting positions and wall interaction along with the spray or drop trajectory have a strong influence on deposit formation (Jain et al. 2017).

Some of the above studies have been done for urea decomposition in open-air conditions and for a fixed mass of urea (Schaber et al. 2004). But, when UWS droplets are sprayed into flowing exhaust gas, the phenomenon is different. Further, studies of deposit formation mainly involve infrared (IR) spectroscopy, ultraviolet (UV)-visible spectroscopy, TGA, and elemental analysis at some particular operating conditions (Weeks et al. 2015; Smith et al. 2016; Brack et al. 2014). The study of parameters affecting the deposit formation, quantification and depletion characteristics in transient load conditions is important. The proper characterization of deposits in transient conditions coupled with depletion characteristics is the complementary information for SCR designers.

It is also found from literature (Börnhorst et al. 2019) that the deposits obtained from a single injection have shown a different trend of their depletion by TGA analysis when compared to that of multi injection. Further, based on wall temperature, the deposits are categorized ( Brack et al. 2016). According to them, in cold operation conditions (*T*_wall_ <150^0^C), a solid form of urea exists. For temperature range, i.e., 150°C < *T*_wall_ < 250°C, the byproducts contain biuret, CYA and ammelide. For higher operating temperatures (*T*_wall_ > 250°C), a low quantity of ammelide forms. Strots et al. (2009) found that there was a drop in the temperature of walls of the SCR mixing chamber for the injected UWS and also, the deposit yield is varying with ambient temperatures. The latter two studies give further insight into the effect of wall temperature on deposits based on the heating cycle of the walls of the SCR mixing chamber that experiences during the transient running condition. When engine load conditions change-over intermittently, fluctuations in temperature take place in the exhaust pipe, which results in changes in the crystallinity of urea deposits, which may lead to a variety of compounds. It is also noted that the complete thermal decomposition of deposits is not possible at the regular exhaust temperature (Shahariar and Lim 2019). When wall temperature drops, the urea depletion rate decreases, and fresh urea deposit forms over the existing layer due to the time delay in achieving steady-state temperature. Any idle or delay period before the next injection not only cools the wall but also changes the interface for the freshly injected UWS. This leads to a condition of differential solidification of the deposits. An extra quantity of UWS by fresh injection may lead to introduction of new layer of deposits since there is no sudden rise of temperature of the same in the absence of metal contact. Also, it is noted that it misses out partly the after-impact processes like a rebound and thermal breakup. Heat transfer to impinging droplets and liquid film evaporation results in local cooling of the wall in the regions of liquid/wall interface (Börnhorst et al. 2020). Authors opine, these characteristics are time-variant and also different when there is an existing layer of deposits and UWS injection takes place over it continuously. Hence, subsequent growth stages are different. This leads to further insight into the effect of heating modes on deposit formation characteristics.

In actual SCR systems, if the deposit forms during low exhaust temperature at an idle run, they may deplete at a higher temperature when load condition changes. This alters the NH_3_ inflow to the SCR catalyst. Additionally, the deposits at temperatures 170–200°C are semisolid or liquid in nature which may be driven out of the UWS impaction region during high-velocity conditions. At this condition, the influence of fresh droplets on existing deposits is minimal. This prevails the condition of aging at higher temperatures with a metal contact. Further, in the case of excess deposits in the spray impacted area, only the top surface undergoes dilution, and the rest of the deposits might be under thermal aging. So, a stepwise study comprising the generation of deposits and depletion/aging at higher temperatures finds its importance, and this kind of study is not found in the literature. With this background, the dosage strategy can be upgraded to avoid NH_3_ imbalance. Further, the morphological changes of the deposits formed at a higher temperature after aging reveals probable growth areas. With this intention, an investigation was proposed to characterize the deposits that form at a low-temperature range, i.e., 150–200°C, and age at 300°C in a hot air test rig, and to highlight its significance in evaluation the deposits quantitatively during transient load cycles for a particular SCR system. Further, study of deposit formation due to different heating modes that occur during intermittent and continuous engine run conditions is also aimed as a part of thermal treatment of deposits.

### Experimental procedure

**Fig. 1:**
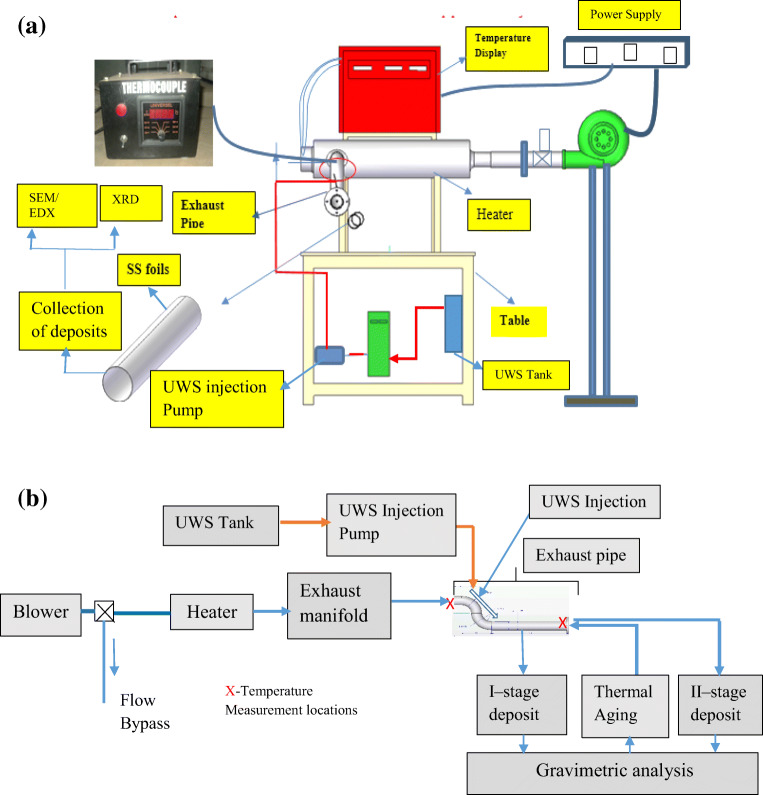
**a** Experimental setup showing UWS injection and deposit formation. **b** Block diagram of the experimental setup

Usually, SCR systems are built on a particular automobile based on several factors like mass flow rate, NO_x_ levels, and space constraints. There is no universal testing instrument to check the performance of the SCR system. However, many of the test conditions cannot be attained by doing tests on engines with SCR attachment. Many researchers (Zheng et al. 2010; Grout et al. 2013) conducted tests in a hot air test rig and obtained the results equivalent to results of the actual SCR unit. So, by revealing this, a deposit formation study using a hot air test rig is found to be a good approximation with actual SCR systems to proceed with the present case. Additionally, the hot air test being simpler and cheaper in construction enables researchers to do repeated test runs. In this context, a hot air test rig is developed as shown in Fig. 1a. The experimental procedure relevant to our investigation is outlined in Fig. 1b.

### Experimental setup

Hot air was used in the experiments, and it was generated in a heater-blower setup. The flow was calibrated for various flow rates with the help of an adjustable valve and an orifice meter setup. The injector was mounted as shown in Fig. 1b over a straight portion of S-bend type stainless steel (SS) pipe of 75 mm internal diameter which delivers air at 0–500 kg/h to match the engine exhaust flow rates of medium to heavy-duty automobiles. Temperatures were sensed in two different locations using thermocouples. UWS was injected for 20 min with an overdosed flow of 8 ml/min. Deposits were collected over SS foils which were flexible, and could be inserted and removed intermittently without damaging the structure of the deposits.

### Gravimetric analysis, SEM/EDX, and XRD studies

Deposits were collected over foils inside the pipe after the first 20 min of UWS injection at an exhaust flow rate of 34.74 kg/h. Later, the generated deposits left to age for 30 min without UWS injection at the same exhaust flow rate. A measuring scale with an accuracy level of ±10mg was used for weight measurement of the foils before and after the tests. The three weights, *W*_1_ initial weight of the foil, *W*_2_ final weight of foil with deposits after UWS injection for 20 min. (pre-age), and *W*_3_ weight of the foil after 30 min. of aging (post-age) conditions, were measured. The weight of the deposit after pre-age treatment was (*W*_2_-*W*_1_) and the weight of the deposit after aging was (*W*_3_-*W*_1_).

Scanning electron microscope (SEM) uses a focused electron probe for structural study and chemical information from a region of interest. The high resolution of SEM makes it most suitable to characterize specimens in nm to μm scale. The instrument used for SEM/EDX (EVO MA18 with Oxford EDS(X-act)) works in the air-conditioned environment (21–24°C) at relative humidity is less than 60%, with a nitrogen supply of 99% purity and regulated at 0–3 bar. It has a magnification range of 1X–1,00,000X and required resolution (3 nm at 30 kV, 2 nm at 3 kV, 15 nm at 1 kV and 4 nm (low vacuum, 30 kV)). EVO 18 operates with the secondary electron (SE), backscattered electron (BSD) and in variable pressure (VP) modes. EDS detector quantifies the elements present in the sample. The attached EDS has the capacity of nine sample holders with a magnification of ×1000 and aperture size of 100 microns. Table 1 gives the probable compounds of urea depletion and their composition for comparative study.

**Table 1:** The chemical composition of reference compounds

Compound	C	N	O	H
Urea	20.00	46.65	26.64	6.71
Biuret	23.30	40.77	31.04	4.89
Triuret	24.65	38.25	32.87	4.12
Cyanuric acid (CYA)	27.91	32.56	37.19	2.34
Ammelide	28.13	43.75	24.98	3.15
Ammeline	28.34	55.11	12.59	3.96
Melamine	28.56	66.64	0	4.79

XRD analysis was done using powder XRD with a voltage of 20–40V and current of 2–15mA along with chiller water circulation. Once the detector rotates through their respective angles, intensities of diffracted X-rays are recorded continuously. The swiveling angle 2θ varies from 5 to 110° with a scan speed of 1°/min-8°/min. More details of XRD fundamentals are found in the literature (Bish and Post 1989; Cullity 1978; Klug and Alexander 1974).

**Fig. 2:**
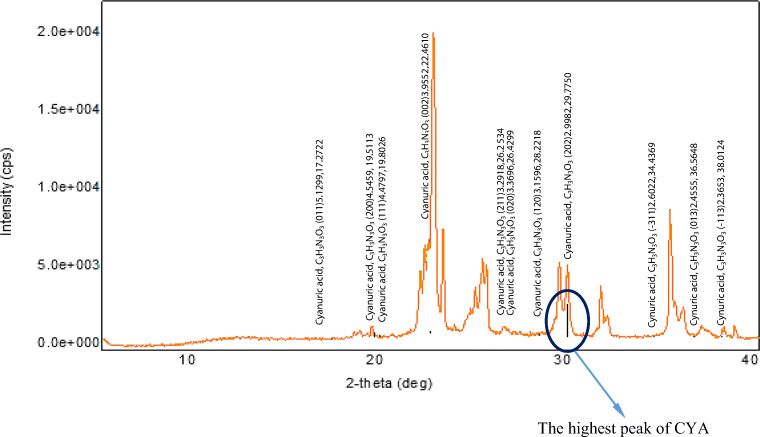
Comparison of XRD pattern of tested sample at condition showing the presence CYA

The standard Joint Committee on Powder Diffraction Standards (JCPDS) files were referred and peaks were compared starting from the highest intensity peaks of the detection compounds (CYA, ammelide, ammeline, biuret, triuret, etc.). The highest peak of the XRD pattern of the detection compound is merged with the XRD of the tested sample. If there are any peaks in the region of 2θ that corresponds to the highest peak of detection compounds, then it is an indication of the presence of a particular detection compound in the tested samples. The details of 2θ for the highest peaks of detection compounds are found in Table 3 along with JCPDS file numbers. A typical example of the detection of CYA is shown in Fig. 2.

## Results and discussion

### Gravimetric analysis for pre-age and post-age conditions

With the objective of identifying the nature of deposits and their chemical variation at higher exhaust gas temperatures, deposits collected at specified lower temperatures were kept for aging at elevated temperature 300°C. This resembles a prevailing condition in actual engine exhaust when load changes from idle to part loads. In actual SCR run condition, there is an injection of UWS when running at a higher temperature to overcome NO_x_. This leads to dilution of the top surface of the deposits. On the contrary, injection of UWS also cools the wall continuously, which might enhance the deposit formation. Further, it is mentioned in the literature that the TGA studies with 5% water content hardly have their effects on the decomposition of urea for prolonged exposure 100–250°C (Zhang et al. 2017). So, aging in the absence of water or without UWS injection is nearly an approximation to deposit aging during actual running conditions with stoichiometric injection of UWS at higher temperatures. Also, during entrainment of the droplets at higher flow rates, the deposits form in areas other than impaction, and those areas are less prone to dilution by the injected UWS. In such cases, the deposits at those locations are under the state of thermal aging.

By identifying the above scenario, thermal aging was extensively studied to analyze the deposits due to variation in temperature of exhaust gases when the load changes from idle to part loads. Accordingly, the deposits formed at lower temperatures 150°C, 175°C, and 200°C (pre-age temperatures) were taken for thermal aging at 300°C. From the earlier studies by the authors (Sadashiva Prabhu et al. 2017), the deposits were found to be higher at lower flow rates. Likewise, we have chosen a lower flow rate of 34.74 kg/h. Further, continuous injection of UWS to exhaust gas yields lesser deposits as there is no cooling cycle during the process. However, we followed the continuous injection for 20 min but increased the dosage up to 8 ml/min to compensate this effect. Accordingly, the deposits were collected in the hot air test bench (Fig. 1a) at a dosage of 8 ml/min with a hot air flow rate of 34.74 kg/h for the above three temperatures at an injection angle of 30°. The gravimetric analysis for both pre-age and post-age conditions was done, and the results are tabulated in Table 2.

**Table 2:** Gravimetric test results in pre-age and post-age conditions

Temperature (°C)	Weight of deposit after 20 mins during pre-age (*W*_2_-*W*_1_) g **A**	Pre-age deposit formation factor **α %**	Weight of deposit after 30 mins of aging at temperature 300°C (*W*_3_-*W*_1_) g **B**	Remain rate of deposits β% (*W*_3_-*W*_1_) ×100/(*W*_2_-*W*_1_) **B×100/A**	Deposit consumption rate (rate of depletion(*k*)) of pre-age deposits during aging, (*W*_2_-*W*_3_/*t* ) g/min for time *t* (**A-B)/30**
150	16.97	30.21	1.14	6.7	0.5277
175	14.75	26.26	0.66	4.47	0.4697
200	12.55	22.34	0.49	2.7	0.402

Results obtained from the three pre-age conditions are compared with the results of the gravimetric analysis done at 150°C (Sadashiva Prabhu et al. 2017). The risk of deposit formation is evaluated using pre-age deposit formation factor *α* (*α* =deposit quantity×100/(0.325×total injected UWS for 20 min)), which is lower (i.e., 30.21%) when compared with that of intermittent run for 20 min (i.e., 57.69%) (Sadashiva Prabhu et al. 2017) even though the dosage was doubled in the present case. A similar trend was observed even at 200°C.

It is noted that pre-age deposit formation factor is in the range 22–30% for the temperature range 150–200°C showing a declining trend with pre-age temperature. Further, the remain rate (β) after aging are in the range 2–6.7% undergoing noticeable transformation of deposits. The final mass of deposits after aging is found to be higher for the deposits formed at lower pre-age temperature 150°C, compared to the other two cases (175°C and 200°C). Authors opine this is due to increased initial mass of deposits during pre-age (i.e., 16.97 g, which is higher compared to other two cases). But, the time-dependent deposit consumption or depletion rate (*k*) during 30 min of aging is found to be decreasing progressively with increase in pre-age temperature. This analysis of results infers that deposits that form at lower pre-age temperatures higher quantitatively. But, its depletion occurs at a faster rate during aging, whereas the deposits formed at higher pre-age temperatures lead to lesser deposits, but they deplete at a slower rate during aging. These variations of depletion rates are emphasized and shown using lines AA1, BB1, and CC1 in Fig. 9 (“Significance of the study on deposit formation and depletion characteristics” section). This kind of analytical observation leads to the inference that deposits formed at higher pre-age temperatures are less prone to deplete during aging at higher temperature, which is noteworthy during quantitative deposit prediction and UWS dosage strategy.

**Fig. 3:**
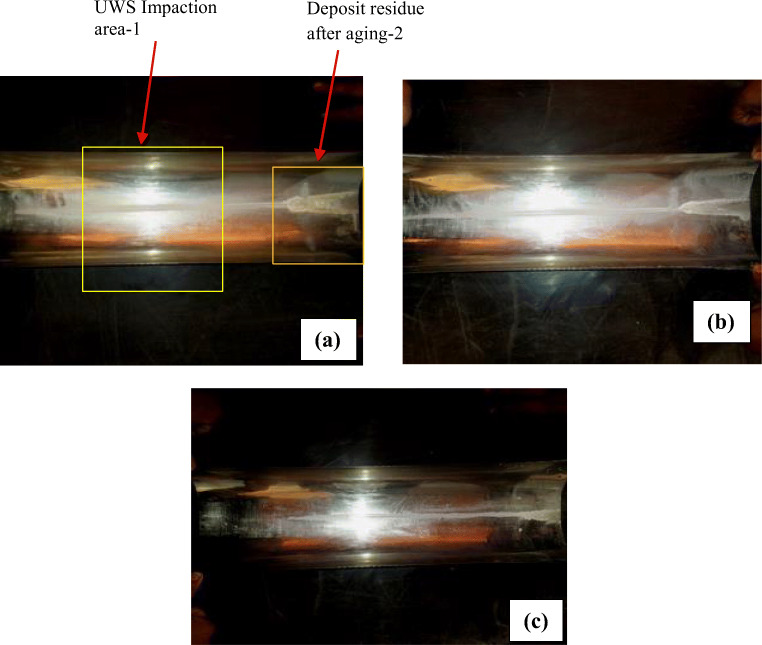
Images of deposits aged at 300°C for conditions. **a** Pre-age 150°C. **b** Pre-age 175°C. **c** Pre-age 200°C

From physical observation (Fig. 3a–c) and tabulated values, it is inferred that conversion percentages of deposits are higher for the deposits formed at lower pre-age temperatures as the quantity was in excess. In such cases, the dosage strategy is to be tuned at high temperatures to avoid NH_3_ leakage due to depletion of urea deposits. The amount of deposits and NH_3_ leakage after depletion of deposits depends on the duration to which the low temperature prevails for exhaust gases

### Physical observation of deposits after aging

The physical nature and morphology of deposits were studied for deposits collected after the initial 20 min. Deposits spread across nearly 1/3 lower circumference with an increased thickness at the periphery of UWS in the impaction area and resemble some of the cases narrated in the earlier work of authors (Sadashiva Prabhu et al. 2017). However, minor variations were created due to variation in dosage and difference in injection strategy. So, the emphasis was given to post-age conditions as there was a substantial decrease in the quantity of deposits. The final nature of aged deposits of three pre-age conditions is shown in Fig. 3a–c.

The phenomenon involved in aging and subsequent depletion proceeds without liquefaction by fresh UWS. As urea melts and depletes, it forms liquid due to rise in temperature during aging, and thereby, the natural agglomeration and settling by gravity was observed for all cases (Fig. 3a, b, c). Additionally, liquid urea gains momentum as surface shear prevails due to incoming exhaust gas flow. In the first case, for the deposit of pre-age temperature 150°C (Fig. 3a), the urea being in larger mass exposed to 300°C, resulted in more residual mass and its surface turning pale reddish-yellow. However, this effect was also found in the other two pre-age samples (175°C and 200°C; Fig. 3b and c), but the quantity of deposits was lower. Further, if droplets were driven to the side surfaces of the foil by turbulence, then they transform into some tiny sticky deposits if they are under prolonged exposure during pre-age itself. Such deposits do not deplete at a faster rate when they are aged. Instead, they form some complex sticky compounds on side surfaces. This phenomenon is prone to exhibit for higher pre-age temperatures. Besides, reddish-yellow deposit mass was found in some sidewall locations, indicating the presence of deposits that do not melt even after 30 min of aging.

### Elemental analysis using EDX

**Fig. 4:**
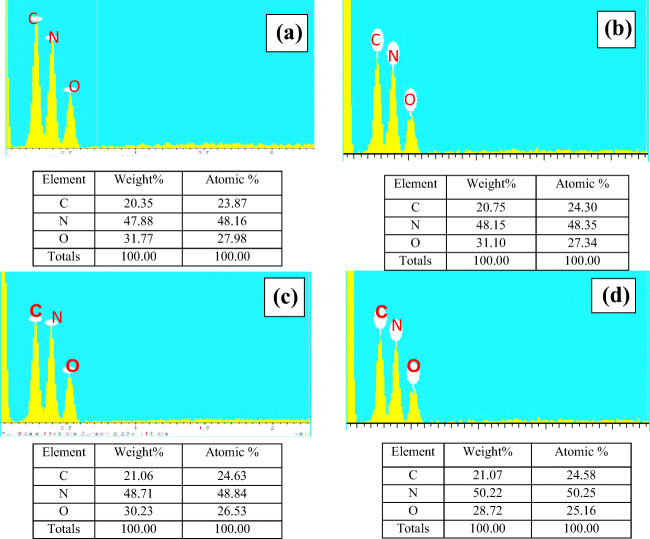
The elemental analysis of samples with conditions. **a** Pure urea. **b** Pre-age 150°C. **c** Pre-age 175°C. **d** Pre-age 200°C

The surfaces of the deposits were analyzed for compositional variation. Figure 4 indicates the EDX results of original urea (Fig. 4a) and pre-age samples (Fig. 4b, c, d), showing percentages of carbon, nitrogen and oxygen. Referring to Table 1, the percentage of carbon increases for all compounds and that of nitrogen increases for ammeline and melamine when compared with the respective carbon percentage of original urea. Further, percentage of oxygen is found to be higher for biuret, triuret, and CYA when compared to that of original urea. These are the preliminary observations to inspect the changes in crystallinity and composition. For the sample with pre-age condition 150°C, the amount of both carbon and nitrogen increased to 20.75% and 48.15% respectively from their original weight percentages. This gives the inference that urea would have turned to any other types of compounds given in Table 1. As percentage changes are small, the appearance of other constituents can be in lesser quantities. For the samples with pre-age condition of 175°C, the weight percentages of carbon and nitrogen increased and that of oxygen decreased from the respective values of original urea. This shows the presence of CYA slightly. For the sample with pre-age condition of 200°C, nitrogen increased by 2.34%, and oxygen decreased approximately by 3.05% from that of pure urea, indicating the presence of biuret or ammeline. However, changes are minimal, so the majority of the contents can considered as urea in all three cases. With the above inference, the urea transformation known to be sluggish is due to low temperature and over-dosage. So, further investigation is done using XRD to identify the traces of various other possible compounds, which is explained in subsequent sections.

### XRD analysis

As we observed through EDX analysis, it is not possible to predict the compound exactly by noting the changes with their carbon, nitrogen, and oxygen percentages. Further, compositions locally vary. The samples after 1^st^ stage (pre-age) and 2^nd^ stage were analyzed through XRD to check the variation in crystallinity and the presence of various byproducts with respect to rising temperature. The procedure is detailed in the “Gravimetric analysis, SEM/EDX, and XRD studies” section.

#### Pre-age analysis for temperature range 150–200°C

**Fig. 5:**
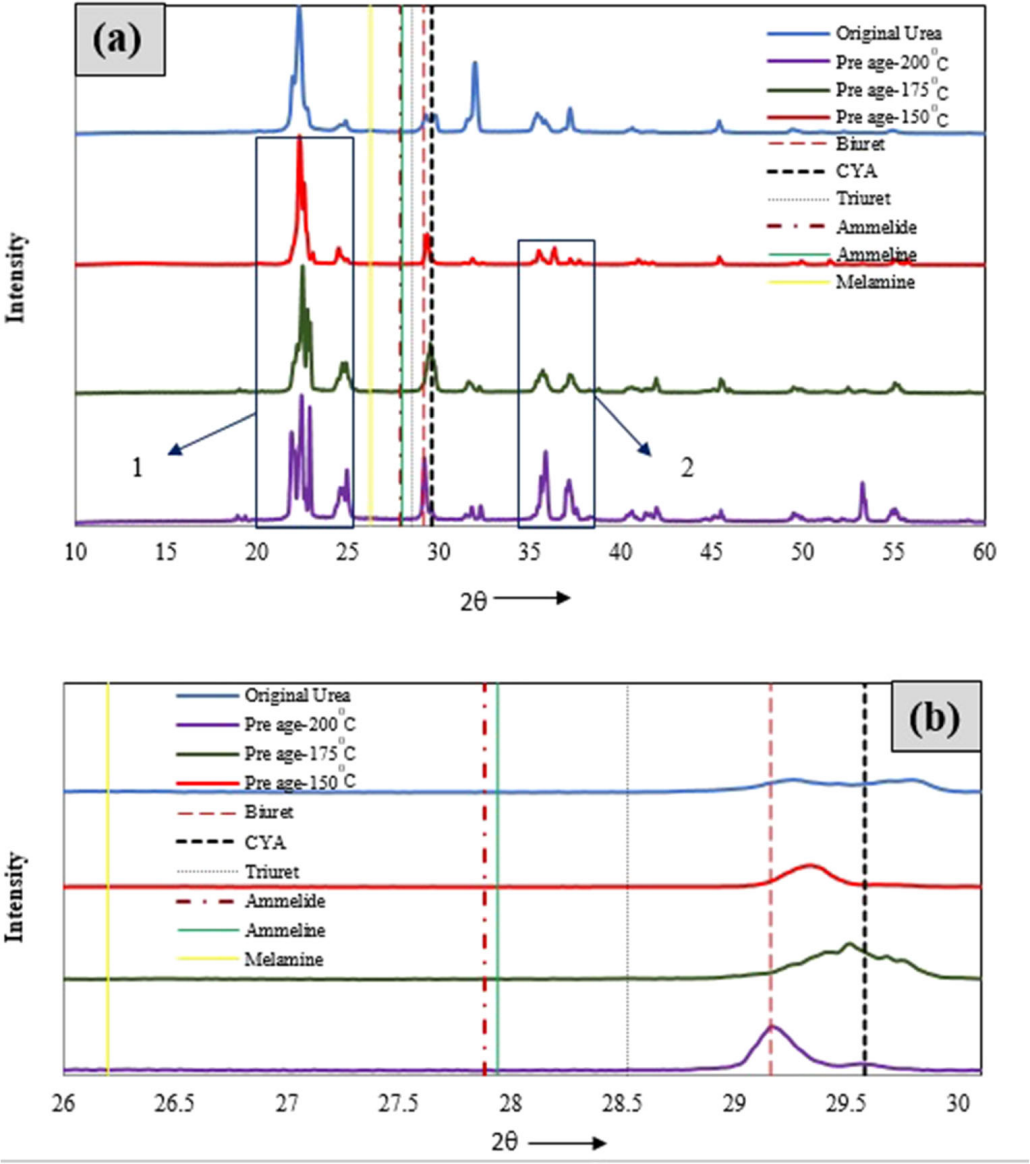
**a** The comparison XRD results of aged samples at different pre-age conditions. **b** Magnified view at 26–30°

The pre-age samples tested at 150°C, 175°C, and 200°C were taken to XRD analysis initially. The intensity vs. 2θ plots are drawn based on results and depicted in Fig. 5a. The 2θ values corresponding to the maximum intensity of various detection compounds (as listed in Table 1) are also plotted, and they are represented as vertical lines. The detection of various byproducts is done according to the procedure shown in Fig. 2. Substantial variation is found with the crystallinity for the three samples showing the effect of varying temperature on deposit formation and its depletion. The major cause for variation in quantity and chemical nature is varying circumferential evaporation and subsequent depletion of solidified urea from wall film formed after UWS impaction. This is caused due to varying exhaust gas and wall temperatures. The drastic variations in XRD peaks are observed for the three pre-age conditions when compared to pure urea, and they are highlighted at locations 1 and 2 in Fig. 5a. However, the maximum intensity peaks lie at 22.15°, in all the cases, revealing non-transformations of urea, leading to its deposits. Additionally, there are peaks at 29.1593° and 29.78° with increasing/varying intensity with respect to pre-age temperatures. These characteristic peaks show the presence of biuret and CYA along with urea for all the three pre-age conditions with differences in their intensities (Fig. 5a, b). In support of this, the EDX results of these cases revealed no much variation in C, H, and O weight percentages as described in the “Elemental analysis using EDX” section.

There are remarkable changes at 200°C when compared to the other two cases as observed through respective XRD patterns. This is due to the fact that at above 190°C, biuret was not stable. Most of the byproducts like CYA, ammelide, and ammeline start forming from the decomposition of biuret above 190°C. Further, urea transforms to liquid at 200°C due to higher convection heat transfer, and the extent of melting is decided by residence time and UWS dosage. Additionally, biuret formed at that temperature forms an eutectic mixture and exhibits liquid nature. This liquid solidifies and leads to a change in crystallinity for solidified deposit. So, remarkable changes are observed for 200°C with the crystallinity shown in urea characteristic 2θ region 22–23° indicating verge of transformation of urea to other related compounds (Fig. 5a). The appearance of various compounds for the above pre-age conditions are further analyzed by showing XRD patterns in the range 26–30° in a magnified view, as shown in Fig. 5b. The biuret and CYA are observed in addition to urea for all the cases, as some of the peaks fall in the characteristic 2θ region (29.1593–29.58°). It is noted that there are no peaks in characteristic 2θ regions of melamine, ammeline, ammelide, and triuret indicating their absence in all three pre-age deposits. This is due to the fact that their reaction rates corresponding to the formation of these compounds are low at temperatures below 200^0^C, and their transformation is sluggish even when UWS dosage is excess.

The results obtained are compared qualitatively with experimental observations found in literature for nearer operating temperatures (with different ratio of UWS/mass flow rate of exhaust gas), between temperatures 150°C and 200°C (Börnhorst et al. 2019). According to literature, byproducts like urea and biuret found at 150°C, and, triuret and CYA are the additional byproducts at 190°C. But, in the present cases, most of the deposits are urea and small fractions of biuret and CYA without the presence of any triuret. It is seen from the literature (Zhang et al. 2017) that the depletion and decomposition temperatures for biuret are 190°C and 193°C, respectively. The biuret quantity being minimal for the case 175°C and 200°C it infers that urea transforming to biuret is minimal when the dosage is excess, and also biuret forms out urea in smaller fractions is also unstable. The biuret formed lower to 190°C may transform to CYA with the presence of HNCO for the prevailed condition. In support of this, the comparison of TGA of urea and urea-related compounds shows biuret decomposes in early stages (Zhang et al. 2017).

#### Post-age analysis

During aging conditions, deposits undergo continued pyrolysis owing to the formation of byproducts that are typically observed at higher exhaust temperatures in usual SCR. In contrast to TGA studies of urea and urea pyrolysis in an open-reactor vessel, there was no effect on the vapor pressure of vapors produced during depletion in the present case of aging, since they were carried away along with flowing exhaust. Further, convective heat transfer to deposits and expulsion of gaseous products of urea depletion leads to differential characteristics to deposits. There was no fresh UWS injection, which leads to a lack of wet surfaces for existing deposits.

**Fig. 6:**
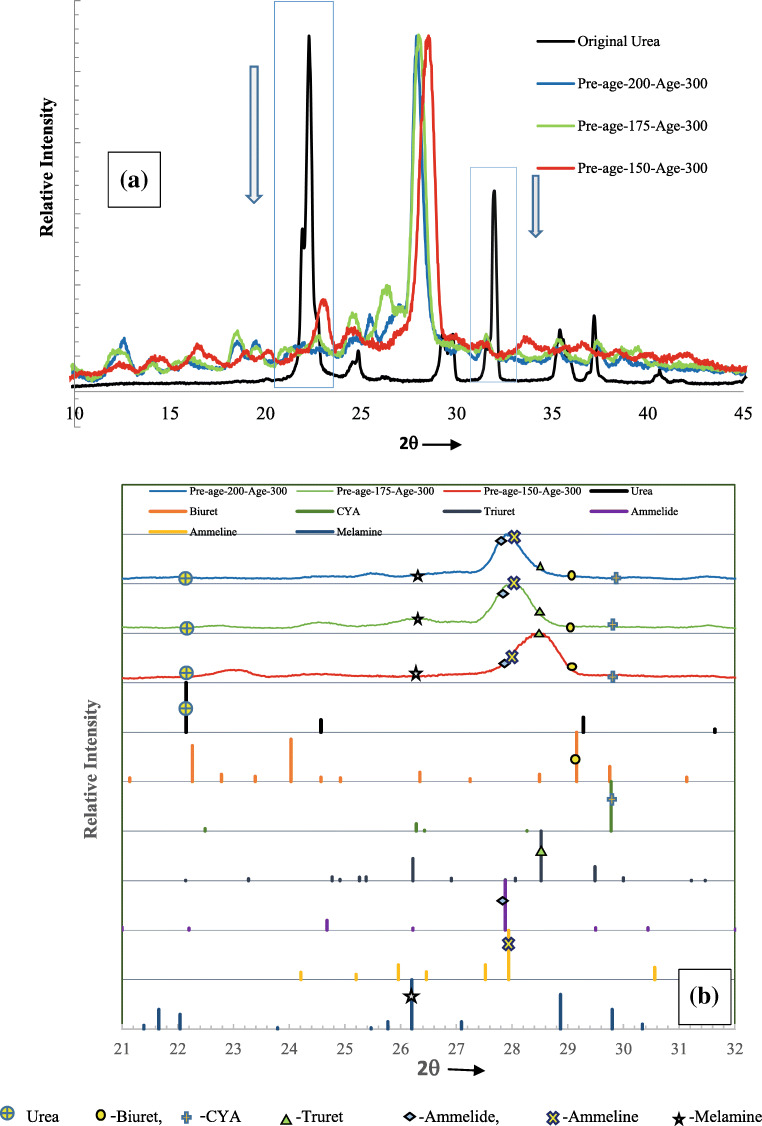
The comparison of XRD results of urea and aged samples of different pre-age conditions. **a** Comparison of XRD patterns. **b** Identification of various compounds

XRD analysis was done for aged samples to identify the change in the composition and crystallinity compared to that of pure urea, and the results are shown in Fig. 6a. For all samples undergone aging treatment at 300°C reveal that there is no urea left in the sample as there were no stronger urea-related XRD peaks for a 2θ angle of 22.15° (regions are shown in boxes, and arrows show characteristic peaks being lost in Fig. 6a). In order to differentiate compounds formed, the XRD patterns are compared with the highest peaks of detection compounds. The results are presented in Fig. 6b. Table 3 gives the data of the JCPDS file name, 2θ values, d-spacing of standard byproduct species, and the presence of them in aged samples.

**Table 3:** Comparison of presence of various urea-related compounds in the tested samples aged at 300°C

Compound	JCPDS file no	“2θ” Highest peak	“d” A^°^	Pre-age 150°C, age 300°C	Pre-age 175°C, age 300°C	Pre-age 200°C, age 300°C
Ammelide	00-031-1527	27.88	3.197	Yes	Yes	Yes
Ammeline	00-031-1526	27.94	3.19	Yes	Yes	Yes
Biuret	00-038-1614	29.1593	3.06	Low intensity	Low intensity	Low intensity
CYA	00-023-1637	29.78	2.998	Low intensity	Low intensity	Low intensity
Melamine	00-024-1654	26.2	3.399	No	Yes, with lower intensity	Yes, with lower intensity
Triuret	01-072-1631	28.52	3.127	Yes	Low intensity	Low intensity
Urea	00-008-0822	22.15	4.010	Very low intensity	Very low intensity	Very low intensity

The aged sample with pre-age condition of 150°C has shown the presence of triuret along with the traces of biuret, CYA, ammelide, and ammeline. This shows that the alternate routes of the transformation of biuret formed out of urea. The usual route of decomposition of biuret to CYA and then to HNCO results in no deposit in the sample. But in the present case, urea being to a larger extent in pre-age stage is believed to decompose into usual NH_3_ and HNCO giving out biuret or triuret to smaller extents. The HNCO formed in this stage would have supported the transformation of some part of biuret to triuret rather than converting itself into CYA and then to HNCO during aging.

The aged samples of 175°C and 200°C, XRD results reveal the presence of ammelide and ammeline (characteristic 2θ angles 27.88° and 27.94°). This clearly shows the transformation of urea to biuret and continued CYA decomposition. Further, biuret transformation into ammelide and ammeline when deposits aged at 300°C in the absence of UWS is noticed substantially. This is supported by HNCO formed from the depletion of urea deposits. In addition, CYA conversion leads to the liberation of water results in ammelide. Authors opine that the absence of water without UWS injection also supported this conversion. CYA was the major byproduct when tested at 300°C at a flow rate of 100 kg/h (Liao et al. 2017) with the presence of UWS. But, the present study does not reveal the presence of CYA for the same temperature. It could also be the possibility that the minor pre-age deposit, biuret, directly transformed into ammeline and ammelide when the temperature is raised to 300°C. Additionally, there are also chances of urea directly being transformed to ammelide in the presence of HNCO, which may form due to depletion of urea deposit. Melamine, being a lesser quantity, has shown lower intensity (2θ angle 26.2°) in the XRD of aged samples of pre-age temperatures 175°C and 200°C (Fig. 6b). But, only traces of CYA are seen without any high-intensity peaks. The byproducts formed at low temperatures such as CYA and Biuret are not observed with higher intensity peaks for all three aged samples.

The results of aging are compared with test results in the literature (Börnhorst et al. 2019; Liao et al. 2017) for temperature range of 280–320°C (with different exhaust mass flow rates and UWS injection) that showed the presence of CYA substantially. But, in our cases, ammelide, ammeline and triuret are the major deposits, and, the appearances of biuret and CYA are meek. The aging was carried out at 300°C without any UWS injection. This results in a lack of dilution of deposits resulting in the evolution of byproducts, which are generally known to appear at higher temperatures. In support of the study, it quoted in literature (Börnhorst et al. 2019) due to dilution by injected UWS, deposits deplete. Accordingly, the authors opine that the aging effect is minimized when UWS injection persists. In the present case, aging progressed for a 30 min without dilution. This infers that long-term aging of those deposits without dilution leads to byproducts that likely to appear at high temperatures in usual SCR condition with same exhaust temperature. The absence of fresh UWS, the difference in dosage, and flow rate would cause differential deposit formation characteristics. Additionally, the lack of injection during the period of aging reduces the wall-cooling effect, leading to increased heat flux towards deposits; thereby, the byproducts can be different compared to that at regular SCR process with UWS injection and wall cooling.

In actual SCR systems, when exhaust flow rate is high and its temperature is low, the low-temperature deposits (biuret, CYA) may form in the region away from the impaction area due to entrainment, and they may undergo aging with an increase in temperature. The present study on aging can be correlated to the study on long-term deposits, which qualitatively vary with the regions as they get treated differently due to flow rate variation.

### Morphological inspection of urea deposits

**Fig. 7:**
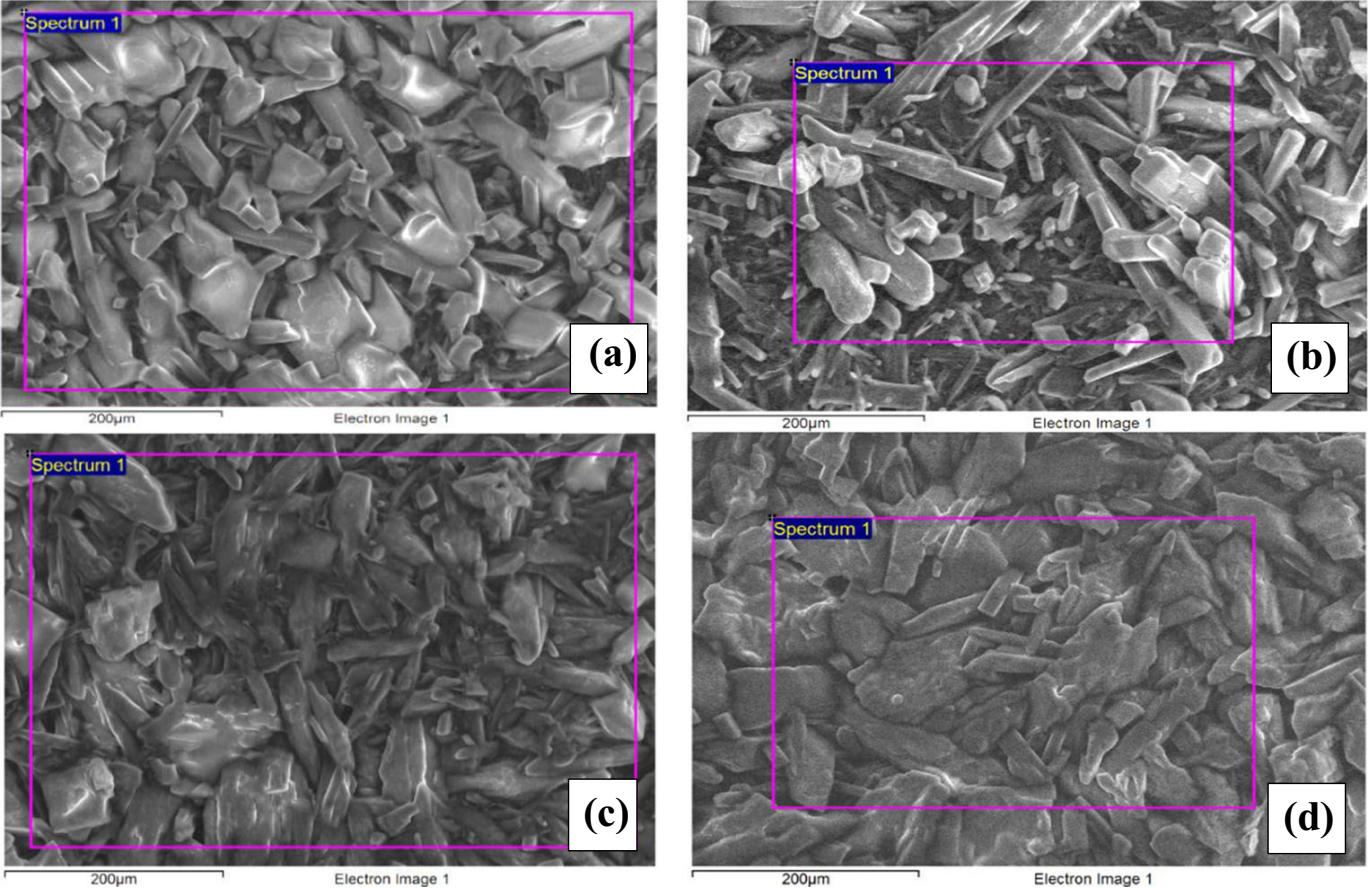
SEM studies of deposits with pre-age conditions. **a** Pure urea, **b** 150°C-34.74 kg/h, **c** 175°C-34.74 kg/h, **d** 200°C-34.74 kg/h

**Fig. 8:**
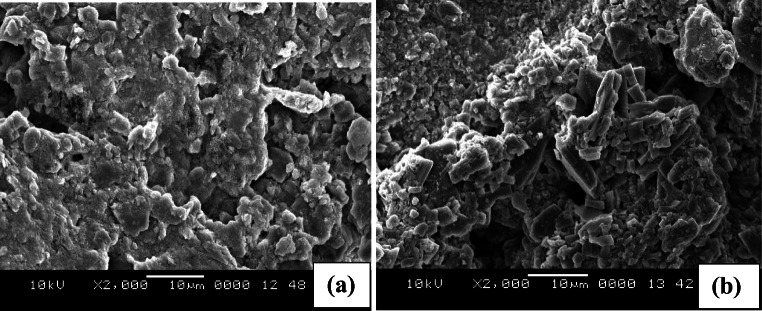
SEM studies of deposits with pre-age conditions. **a** 150°C-34.74 kg/h and **b** 175^0^C-34.74 kg/h and undergone aging treatment at 300°C

The particle size and physical nature of urea deposits find their importance as they are related to the growth of deposit mass. Variation in the porosity of deposits alters the convection heat transfer; thereby, the depletion and aging characteristics of deposits vary with the prior nature of deposits. The morphological variations for the pre-age and post-age conditions are shown in Fig. 7b–d and Fig. 8a, b respectively. The general observation is, for a pre-age sample of 150°C, the deposit resembles pure urea (Fig. 7a, b) and the microstructure shows the urea crystals of irregular or distorted globular shape of intermediate size with smoothened corners. The grains are of a higher aspect ratio and look like columnar. But, for the pre-age sample of 175°C, the grains are slightly different in their shape and size. There are some evolved patches over the surface of each crystal in magnified view. For the pre-age sample of 200°C, the deposits are formed due to the solidification of semisolid/liquid melt. These deposits are found as loosely bonded particles of larger size with lesser aspect ratio in a more dispersed manner.

More structural differences are observed for aged samples during SEM study (Fig. 8). The morphological variations show a more widespread deposit structure without any distinct edges to each particle for the sample of pre-age condition 150°C. The structure apparently looks like it is evolved out of combined and continued growth out of prolonged aging. However, for the samples pre-aged at 175°C, the structure is more distinguished, resembling particle nature. In the sample of pre-age condition 200°C, yellow-brown deposits evolved and they were not analyzed with SEM images as the yield is too low.

### Significance of the study on deposit formation and depletion characteristics

There are many kinetics and numerical prediction methods proposed by authors (Sadashiva Prabhu et al. 2017; Smith et al. 2014; Brack et al. 2016) to streamline and evaluate the deposits quantitatively and also to identify the areas of deposit formation. There are also simulation studies on the development of a mixer, which reduces urea deposits in SCR (Huang et al. 2020). Computational fluid dynamics (CFD) simulation in the development cycle helps in the prediction of deposits under specific spray load conditions, but in dynamic conditions, it has a limitation in prediction (Betageri and Rajagopalan 2016). Numerical methods of obtaining results of deposit formation using CFD codes are time-consuming. In this context, a zero-dimensional model with the perspective of deposit formation characteristics, considering major influencing factors like dosage, temperature flow rate, etc., would fetch data to the proximity at various operating conditions of SCR.

**Fig. 9:**
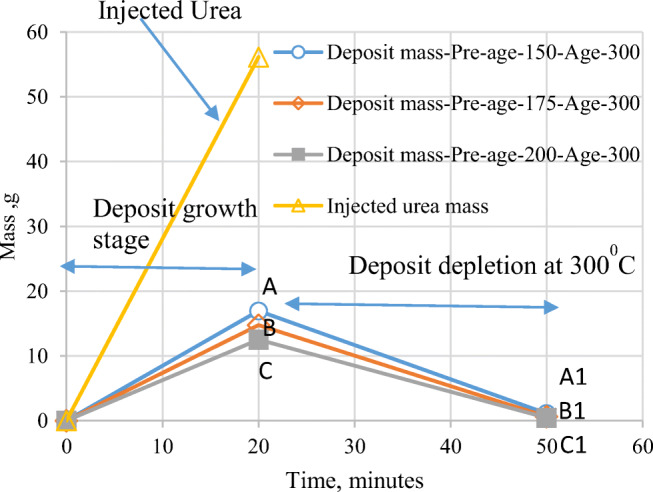
Representation of urea deposits growth during pre-age and depletion after aging

A phenomenological model was proposed by authors in their earlier work (Sadashiva Prabhu et al. 2017) for the formation of the deposits. Smith et al. (2016) found that deposits appeared to grow linearly with time. Likewise, the weights of deposits at 150°C, 175°C, and 200°C after 20 min are marked (points A, B and C) and joined to the origin, with their slopes representing deposit growth rates (Fig. 9). Similarly, weights of the deposits after aging also represented (A1, B1, and C1) and joined to corresponding pre-age points A, B, and C. Now, in the present model, the deposit formation rate can be obtained by taking the slopes of lines OA, OB, and OC and depletion rate by the slopes of lines AA1, BB1, and CC1. Further, it is noteworthy that the slopes are still steeper when urea deposits deplete in the presence of incoming injection of UWS with the presence of water content. The deposit depletion after cold-run or when load changes should be accounted by considering depletion rates. The net deposit accumulation can be obtained by *m*_dep_ = (growth rate-depletion rate) × duration of running. Growth rate and depletion rate are predetermined by repeated trials at particular dosage, temperature, flow rates, etc. The above method of obtaining the amount of deposits would be supplementary information to predict the amount of deposits and can be obtained for any type of diesel-run vehicle. Further, it can also be extended to similar types of SCRs considering transient working conditions using data of deposit formation and depletion at various operating conditions or obtaining data by suitable DOE techniques.

### Characterization of deposits obtained for various heating modes

**Fig. 10:**
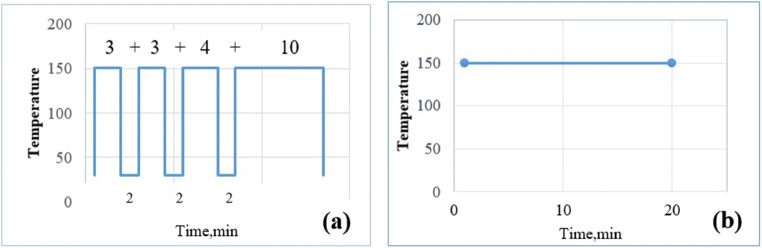
**a** Intermittent heating and UWS Injection. **b** Continuous heating and UWS injection

**Fig. 11:**
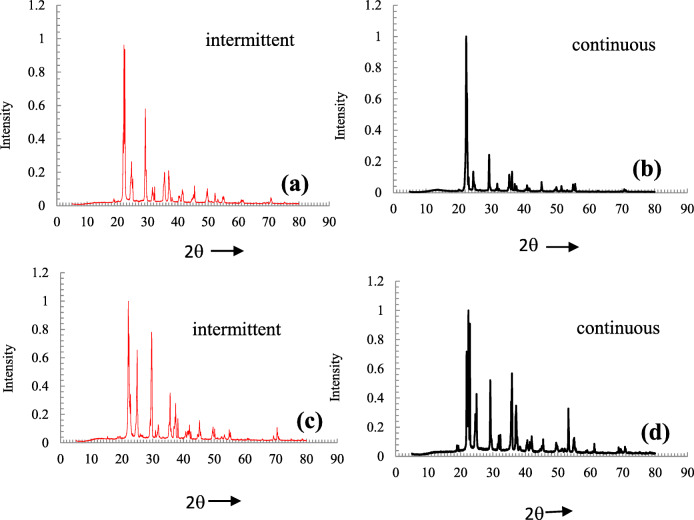
XRD patterns of deposit samples tested at **a** intermittent heating, 150°C; **b** continuous heating, 150°C; **c** intermittent heating, 200°C; **d** continuous heating, 200°C

As mentioned in the “Studies on urea deposit formation” section, it is worthwhile to study the deposit formation considering the effect of intermittent and continuous engine run conditions where the differential thermal treatment is experienced. Similar conditions are achieved in the present setup for temperatures 150°C and 200°C. The intermittent engine-run condition is achieved by a test-run with hot air flow along with UWS injection for cumulative times 3, 6, 10, and 20 min with a delay period of 2 min (Fig. 10a). Continuous engine run condition is achieved by conducting a test run on the same setup with continuous hot air flow and UWS injection for 20 min (Fig. 10b). For the sample tested at 150°C-34.74 kg/h, during intermittent condition, the deposits are greater by quantity due to cooling during delay periods. XRD studies reveal that urea and biuret are constituents in it, with urea being in major fraction (Fig. 11a). The deposits of continuous test run case show the presence of the same urea and biuret (Fig. 11b). While comparing XRD patterns, there are number of additional peaks with different intensities for the former case.

For the samples tested at 200°C-34.74 kg/h, in intermittent condition, the major constituent is urea, but CYA is also found to some extent. It is believed that for intermittent run, the CYA appeared due to a rise in temperature (Fig. 11c), and time delay that leads to the transformation of Biuret to CYA. For the case of a continuous run at 200°C-34.74 kg/h, biuret peaks are observed along with urea-related peaks. In addition, some irregular peaks of various intensities are observed in the urea-characteristic 2θ region (Fig. 11d).

## Conclusion

The deposits obtained at a low-temperature range (150–250°C) in the SCR system of diesel engines exhibit different nature in terms of quality and quantity. When engine exhaust temperature increases due to an increase of load, the deposits tend to deplete. The nature of depletion differs according to the prior condition. To evaluate this, experimental works and followed by characterization were undertaken to generate deposits at low temperatures and to check the condition after aging at 300°C using hot air test rig. The following conclusions were drawn from the study.

- Gravimetric analysis of pre-age and post-age conditions reveal that deposits formed at low temperature deplete at a faster rate and vice versa during aging at high temperature. The study enables us to generate data on the rate of deposit formation and depletion with which a zero-dimensional model could be proposed to find out the amount of deposits for a particular SCR system.
- EDX analysis of the deposits in pre-age temperatures 150–250° has shown that carbon, nitrogen and oxygen percentages are comparable to that of pure urea without any remarkable changes to show traces of other compounds. However, urea, biuret and CYA were observed for pre-age conditions through XRD analysis. Ammelide, ammeline, triuret and melamine were observed for post-age conditions when characterization is done through XRD.
- SEM studies on the morphology and physical nature of the deposits revealed that the grains are different in their shape, size and aspect ratio in pre-age conditions of temperature range 150–250°C. However, drastic variation is observed for aged samples without a specific grain structure.
- The study of deposit formation and depletion characteristics provides additional information to predict the deposit accumulation in SCR system when generating data using CFD simulations are time-consuming.
- Intermittent and continuous test run conditions were introduced to understand the effect of heating cycles. Marginal difference in the quality of deposits is observed through characterization by XRD for intermittent and continuous run cases.

## Supplementary Information

ESM 1 (DOCX 3575 kb)

## Data Availability

All data generated or analyzed during this study are included in this published article
