# Supplementary material for: Effects of thermal treatments on characteristics and morphological variations in the deposits of urea-SCR systems
Source: Environ Sci Pollut Res Int. 2021 Jun 1;28(40):56711–26. doi: 10.1007/s11356-021-14057-4 (PMC8500864; doi:10.1007/s11356-021-14057-4)
Supplement: Supplementary file 1 — (DOCX 3575 kb) [file 11356_2021_14057_MOESM1_ESM.docx]

# Appendix: Survey questionnaire

Thank you for taking the time to read from survey questionnaire to this, your access will bring me great honor. The purpose of this questionnaire is to **understand the respondents' perception of the driving factors for the sustainable development of industrial heritage renovation projects**. The results obtained by the research are **only used for academic research**, and personal information will never be disclosed to the public. This questionnaire is **anonymous**, please feel free to fill in. Your valuable opinion will be the key to the success of this study. Thank you again for your assistance and cooperation. Wish you a happy life.

**Industrial heritage** means sites of industrial culture of historical, technological, social, architectural or scientific value, including buildings, machinery, workshops, workshops, factories and mines, warehouses and warehouses, as well as places for production and use.

**Industrial heritage renovation (IHR)** is the redevelopment and utilization of original industrial buildings. It is a construction method that fully or partially utilizes the original building material entities and retains the historical and cultural content that they carry on the premise that the original buildings are not completely demolished. IHR is a holistic strategy, which includes appropriate protection, restoration, renovation and renovation to some extent. Its core idea is to reanimate old industrial buildings on the basis of meeting the overall development goals of social economy and culture. For example, Beijing Shougang Industrial Park and Xi'an Old Steel Factory Creative Park are all successful IHR cases.

| 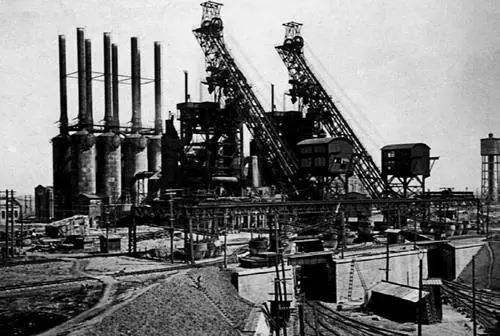 | 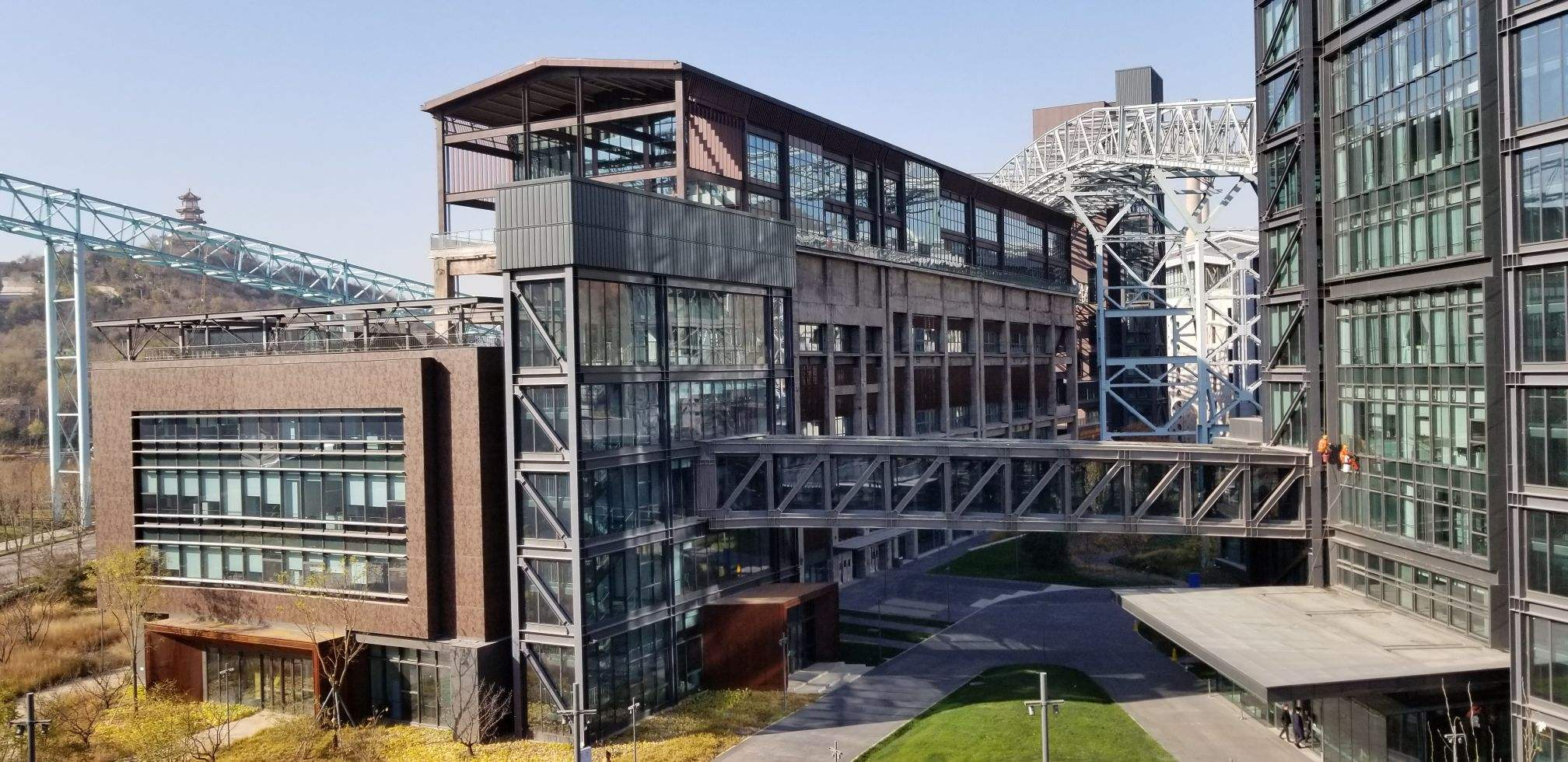 |
| --- | --- |
| Fig. 1 Shougang Group (founded in 1919) | Fig.2 Shougang Industrial Park (reconstructed in 2017) |
| 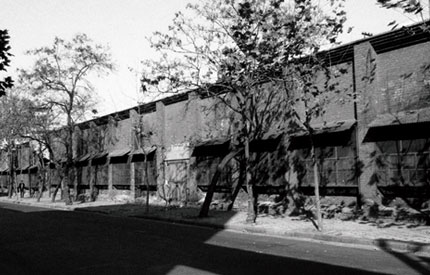 | 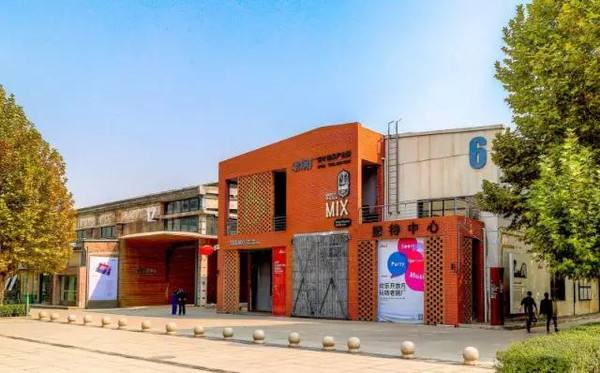 |
| Fig. 3 Shaanxi Steel Works (founded in 1958) | Fig.4 Old Steel Factory Creative Park (reconstructed in 2002) |

Please evaluate the correlation between sustainability and driving factors on a scale of 1 to 5, with "1" representing the least relevant and "5" the most relevant. And tick the appropriate box according to your actual feeling.

| **No.** | **Variables** | **Importance** | | | | |
| --- | --- | --- | --- | --- | --- | --- |
|  |  | 1 | 2 | 3 | 4 | 5 |
| 1 | Repair and maintenance costs | □ | □ | □ | □ | □ |
| 2 | Investment in fixed assets | □ | □ | □ | □ | □ |
| 3 | R&D investment | □ | □ | □ | □ | □ |
| 4 | Increasing investment in sustainable technology | □ | □ | □ | □ | □ |
| 5 | Urban planning and construction | □ | □ | □ | □ | □ |
| 6 | Soundness of laws and regulations | □ | □ | □ | □ | □ |
| 7 | Standardization degree of construction market system | □ | □ | □ | □ | □ |
| 8 | Development status of the construction industry | □ | □ | □ | □ | □ |
| 9 | Construction industrialization level | □ | □ | □ | □ | □ |
| 10 | Government support | □ | □ | □ | □ | □ |
| 11 | Utilization of existing materials | □ | □ | □ | □ | □ |
| 12 | Utilization of existing energy | □ | □ | □ | □ | □ |
| 13 | Water resources recycling | □ | □ | □ | □ | □ |
| 14 | Utilization of construction land | □ | □ | □ | □ | □ |
| 15 | Utilization of solid waste | □ | □ | □ | □ | □ |
| 16 | Construction quality | □ | □ | □ | □ | □ |
| 17 | Engineering management level | □ | □ | □ | □ | □ |
| 18 | Construction mechanization level | □ | □ | □ | □ | □ |
| 19 | Labor productivity | □ | □ | □ | □ | □ |
| 20 | Cultural heritage protection capability | □ | □ | □ | □ | □ |
| 21 | Public service capacity | □ | □ | □ | □ | □ |
| 22 | Comfort level for residents | □ | □ | □ | □ | □ |
| 23 | Architectural design level | □ | □ | □ | □ | □ |
| 24 | Suitability level of development model | □ | □ | □ | □ | □ |
| 25 | Completeness of supporting facilities | □ | □ | □ | □ | □ |

Note: If necessary, a detailed explanation is at the end of the text.

**If you have any other suggestions or opinions, please write below:**

| **Basic Information** | | | | | |
| --- | --- | --- | --- | --- | --- |
| **1. Your gender** | □Male | □Female | **3. Your city** |  | |
| **2. Your age** | □＜18 | □18~30 | □31~40 | □41~50 | □＞50 |
| **4. Level of education** | □junior high and below □senior high school  □undergraduate college □Master □Doctor | | | | |
| **5.Your company/university/institute** | | □real estate agency □design organization | | | |
| □supervising unit □construction organization □universities or research institutes | | | | | |
| □governmental agencies □consultative machinery □original industrial enterprise | | | | | |

**Thank you!**

1 Repair and maintenance costs refer to the labor, materials and expenses incurred in the maintenance of buildings and various equipment.

2 Investment in fixed assets is the amount of work that an enterprise has to construct and purchase fixed assets in a certain period of time expressed in currency. Including real estate, buildings, machinery, machinery, transportation tools, and enterprises for capital construction, renovation, overhaul and other fixed asset investment.

3 R&D investment is not only an input to support scientific and technological activities, but also a productive input. It includes three parts: research and development activities, transformation and application of scientific and technological achievements, and scientific and technological service activities.

4 The sustainable technology investment increment refers to the establishment of a specialized sustainable development management institution and the formulation of a scientific and reasonable sustainable development management system.

5 Urban planning and construction refers to the comprehensive deployment, specific arrangements and implementation management of urban social and economic development, land use, spatial layout, and various constructions within a certain period of time.

6 Soundness of laws and regulations refers to the effective supervision and management of industrial heritage renovation activities by laws and regulations, maintaining market order, and ensuring the quality and safety of the project.

7 Standardization degree of construction market system reflects the implementation of personnel qualification management, implementation of construction permits, investigation of violations of laws and regulations, dynamic supervision of qualification qualifications, personal credit management of enterprises, and implementation of the completion acceptance filing system.

8 Development status of the construction industry reflects the diversity and specialization of fields, the application of big data and artificial intelligence, financial capabilities and operational vitality, market access system and enterprise credit system qualifications, and the balance of labor and production relations.

9 Construction industrialization level refers to the use of modern management models, standardized architectural design, and modular and factory-based parts production to realize the generalization of building components and the assembly and mechanization of on-site construction.

10 Government support includes fiscal appropriation, fiscal discount interest, tax refund, and gratuitous transfer of non-monetary assets.

11 Utilization of existing materials. Make full use of existing building structures, equipment, construction and other materials.

12 Utilization of existing energy. Make full use of exhaust energy recovery system, heat and cold storage system, renewable energy.

13 Water resources recycling. Use water-saving systems, appliances and equipment to rationally recover rainwater and utilize other non-traditional water sources.

14 Utilization of construction land reflects the utilization of the land in the existing building park.

15 Utilization of solid waste. Control pollution to the environment and recover resources from solid waste.

16 Construction quality. During the construction process or the final product, the project meets the requirements of relevant standards or contractual agreements, including the sum of all its obvious and implicit capabilities in terms of safety, use function and durability, and environmental protection.

17 Engineering management level. Carry out project management from multiple aspects such as technology, safety, process and system, and clarify the responsibilities and goals of construction personnel. It is reflected in the reasonable construction management model, emphasis on safe production and civilized construction, and complete supporting facilities construction.

18 Construction mechanization level. The relative scale of using modern construction machinery instead of manual operation in the construction and production process. It reflects the construction technology level and mechanization level of the construction unit.

19 Labor productivity. The efficiency with which the operator produces engineering products during the working period. It is expressed as the ratio of the output or value of a building product to the corresponding amount of labor consumed.

20 Cultural heritage protection capability assesses the extent to which the management department can effectively protect an industrial civilization with protective value.

21 Public service capacity. The building itself and its surroundings can be compatible with multiple functions (catering, shopping, accommodation, office, entertainment, etc.).

22 Comfort level for residents. Strong comfort and good environment during building use.

23 Architectural design level. The architectural design is beautiful and meets the aesthetic needs of the public.

24 Suitability level of development model. Comprehensive consideration of the structural characteristics, historical value, surrounding environment and other factors of the building, with the premise of maximizing the use of existing buildings, determine a reasonable development model.

25 Completeness of supporting facilities. The parking lot, public restroom, property service center and other supporting facilities are fully equipped.
